# Dynamics of host immune responses and a potential function of Trem2^hi^ interstitial macrophages in *Pneumocystis* pneumonia

**DOI:** 10.1186/s12931-024-02709-1

**Published:** 2024-02-05

**Authors:** Hu-Qin Yang, Han Sun, Kang Li, Ming-Ming Shao, Kan Zhai, Zhao-Hui Tong

**Affiliations:** Department of Respiratory and Critical Care Medicine, Beijing Institute of Respiratory Medicine, Beijing Chao-Yang Hospital, Capital Medical University, NO. 8, Gong Ti South Road, Chao yang District, Beijing, 100020 China

**Keywords:** *Pneumocystis* pneumonia, Single-cell RNA sequencing, Interstitial macrophages, Trem2

## Abstract

**Background:**

*Pneumocystis* pneumonia (PCP) is a life-threatening opportunistic fungal infection with a high mortality rate in immunocompromised patients, ranging from 20 to 80%. However, current understanding of the variation in host immune response against *Pneumocystis* across different timepoints is limited.

**Methods:**

In this study, we conducted a time-resolved single-cell RNA sequencing analysis of CD45^+^ cells sorted from lung tissues of mice infected with *Pneumocystis*. The dynamically changes of the number, transcriptome and interaction of multiply immune cell subsets in the process of *Pneumocystis* pneumonia were identified according to bioinformatic analysis. Then, the accumulation of Trem2^hi^ interstitial macrophages after *Pneumocystis* infection was verified by flow cytometry and immunofluorescence. We also investigate the role of Trem2 in resolving the *Pneumocystis* infection by depletion of Trem2 in mouse models.

**Results:**

Our results characterized the CD45^+^ cell composition of lung in mice infected with *Pneumocystis* from 0 to 5 weeks, which revealed a dramatic reconstitution of myeloid compartments and an emergence of PCP-associated macrophage (PAM) following *Pneumocystis* infection. PAM was marked by the high expression of Trem2. We also predicted that PAMs were differentiated from Ly6C^+^ monocytes and interacted with effector CD4^+^ T cell subsets via multiple ligand and receptor pairs. Furthermore, we determine the surface markers of PAMs and validated the presence and expansion of Trem2^hi^ interstitial macrophages in PCP by flow cytometry. PAMs secreted abundant pro-inflammation cytokines, including IL-6, TNF-α, GM-CSF, and IP-10. Moreover, PAMs inhibited the proliferation of T cells, and depletion of Trem2 in mouse lead to reduced fungal burden and decreased lung injury in PCP.

**Conclusion:**

Our study delineated the dynamic transcriptional changes in immune cells and suggests a role for PAMs in PCP, providing a framework for further investigation into PCP’s cellular and molecular basis, which could provide a resource for further discovery of novel therapeutic targets.

**Supplementary Information:**

The online version contains supplementary material available at 10.1186/s12931-024-02709-1.

## Introduction

*Pneumocystis* pneumonia (PCP) remains a life-threatening opportunistic fungal infection in immunocompromised patients, affecting over 500,000 cases per year worldwide [1–3]. Immunity to *Pneumocystis* involves a dynamic interplay between this elusive fungal pathogen and host responses [4–10]. Therefore, much work has concentrated on characterizing the composition and function of immune cells in PCP, implicating multiple immune cell types including macrophages [6, 10], dendritic cells [7], CD4^+^ T cells [8], and B cells [9], which together participate in combating *Pneumocystis*. However, the precise immune mechanisms and the complex interactions between immune cells in PCP remain unclear.

Macrophages are responsible for recognizing and killing both trophozoites and cysts and produce various cytokines and chemokines, which can further activate CD4^+^ and CD8^+^ T cells [4–6]. Macrophages are usually divided into M1 and M2 subsets. M1 macrophages release pro-inflammation cytokines and play a dominant role in anti-fungal immunity, while M2 macrophages express anti-inflammatory cytokines. The balance between M1 and M2 macrophages is critical for the immune response to against *Pneumocystis.* Nandakumar et al. [11] found that an M1 predominant phenotype was observed in immunosuppressed rats, leading to the failure of *Pneumocystis* clearance. In contrast, M2 macrophage polarization was enhanced in immunocompetent hosts and M2 treatment could promote *Pneumocystis* clearing and reduce inflammation. These findings emphasize the importance of investigating the subtypes and function of macrophages. However, A growing number of studies indicate that the phenotype and function of macrophages are very heterogeneous [12–15]. Because the previous studies of macrophages in PCP were based on whole tissue or restricted to a preselected cell type using cell surface markers, it is difficult to identify the diverse subtypes of macrophages that may react differently to disease.

In this study, we employed a mouse model of PCP and performed time-resolved single-cell RNA sequencing (scRNA-seq) of lung immune cells to investigate the host immune response. Our analysis elucidates the dynamic transcription profiles and functional diversity of immune cell subpopulations in the process of *Pneumocystis* infection. Remarkably, PCP-associated macrophages (PAMs) were emerging in PCP, characterized by high triggering receptor expressed on myeloid cells 2 (Trem2) expression, hypersecretion of inflammatory cytokines and inhibiting the proliferation of T cells, specifically dominant in the recovery stage of PCP. Trem2 primarily expressed on the surface of monocyte-macrophage cells, and depletion of Trem2 in mouse lead to reduced fungal burden and decreased lung injury in PCP. These findings may provide novel implications for identifying the mechanism and the function of immune cells in the host when fighting *Pneumocystis* infection.

## Materials and methods

### Mice

Adult male C57BL/6J and severe combined immunodeficiency (SCID) mice aged 6–8 weeks were obtained from Vital River Lab Animal Co., Ltd. (Beijing, China) and were housed under specific-pathogen-free conditions at the Beijing Institute of Respiratory Medicine (Beijing, China). Trem2^−/−^ mice (stock no. 027197), with the C57BL/6 background were purchased from the Jackson Laboratory (Bar Harbor, ME). All procedures were in accordance with the Capital Medical University Animal Care and Use Committee.

### Preparation of mouse model for PCP

*Pneumocystis murina* (American Type Culture Collection, Manassas, VA, USA) was incubated in C.B-17 SCID mice as we previously described [8, 9]. To generate the PCP model, 100 µl phosphate-buffered saline (PBS; Solarbio) which contained 1 × 10^6^*Pneumocystis* cysts, was injected through the trachea of each mouse, while the control mice were transtracheally injected with 100 µl PBS, which we referred to as mice infected with *Pneumocystis* for 0 W. The superior lobe of right lung was isolated for histological analyses using hematoxylin and eosin (H&E) staining to evaluate pathology. The middle lobe of right lung was used to quantify the *Pneumocystis* burden by real-time PCR. The P. murina RNA primers and probes sequence were described as we previously reported.

### Lung tissue processing and flow cytometry

Preparation of cell suspensions followed a recently published protocol [16]. In brief, an overdose of 0.5% pentobarbital was used to anesthetize mice through intraperitoneal injection. Next, collagenase IV (Solarbio) and Dnase I (Sigma) contained in 1 mL complete 1640 medium (Solarbio) with 10% fetal bovine serum (FBS; Hyclone) were infused into the lung through the trachea. Subsequently, the collected lung tissue was chopped with scissors and incubated for 20 min at 37 °C with mild agitation in the 1640 medium with 10% FBS. After filtration through a 40-µm cell strainer (biolegend), the lung homogenate was spun down at 400 g for 6 min and resuspended in ACK buffer (BD) for 15 min on ice to lyse red blood cells.

After washing with PBS, mouse lung cells were stained with fluorochrome-conjugated antibodies using the recommended concentrations according to instructions provided by the manufacturer. Then, the cells were loaded onto FACS Canto II (BD Biosciences, San Jose, CA, USA) and the resulting FACS data were analyzed by FlowJo (version 10.4, TreeStar, Ashland, OR, USA). Table S1 provides the antibodies used in this research.

### Immunostaining and microscopy

Mouse lungs were perfused with PBS and placed in 4% paraformaldehyde for over 24 h fixation before paraffin embedding. Paraffin-embedded tissues were cut into 4 μm sections and the microwave method was used for antigen retrieval. Sections were blocked with goat serum for 20 min at room temperature (RT) and stained with anti-Trem2 (dilution 1:50, rabbit monoclonal, catalog number MA5-31267, Invitrogen) and anti-MerTK (dilution 1:100, rat monoclonal, catalog number 14-5751-82, Invitrogen) for 2 h at 37 °C. Sections were then stained for 1 h at 37 °C with Alexa Fluor 555-conjugated donkey polyclonal secondary antibody to rabbit IgG (H + L) (dilution 1:200, catalog number ab150074, Abcam) and Alexa Fluor 488-conjugated donkey polyclonal secondary antibody to rat IgG (H + L) (dilution 1:200, catalog number ab150153, Abcam). For cell nuclei staining, 4,6-diamidino-2-phenylindole (DAPI) was used for 5 min at RT. Images were acquired on an Olympus BX-43 microscope (Tokyo, Japan) and analyzed using OlyVIA software (version 3.1.1, Olympus, Tokyo, Japan).

### Cytokine and chemokine protein expression

Both Trem2^l^° and Trem2^hi^ IM populations were sorted from infected mice at 4 weeks post-infection, and Trem2^l^° IMs were sorted from uninfected mice without P. murina inoculation. In total, 1 × 10^5^ cells were cultured in 100 ul RPMI containing 10% FBS for 16 h. The concentration of 36 cytokines and chemokines in the culture supernatant were analyzed using Luminex multiplex assays (catalog number EPX360-26092-901, ThermoFisher, Waltham, MA, USA) on a Luminex 200 platform (Luminex Corporation, Austin, TX, USA) in accordance with the manufacturer’s instructions. Data were evaluated using ProcartaPlex Analyst Software (version 1.0, ThermoFisher, Waltham, MA, USA).

### Cell culture and proliferation assay

Naïve CD4^+^ T cells were isolated from the spleens of WT mice by CD4^+^ CD62L^+^ T-cell isolation kit II (Miltenyi Biotec, Aubum, CA). Sorted naïve CD4^+^ T cells were stained with 0.5 µM CellTrace carboxyfluorescein succinimidyl ester (CFSE) cell proliferation reagent (Thermo Fisher Scientific, Bridgewater, NJ) in 37 °C for 10 min and then the reaction was stopped by adding 10 × volumes of PBS and suspended in complete RPMI 1640 medium. Then, naïve CD4^+^ T cells were cultured (3 × 10^5^/µl * 200 µl per well) on anti-CD3 (2 µg/ml) coated 96-well plates in complete RPMI 1640 medium containing anti-CD28 (1 µg/ml).

Both Trem2^l^° IM (CD45^+^ CD64^+^MerTK^+^ CD11b^+^ SiglecF^−^ Trem2^l^°) and Trem2^hi^ IM (CD45^+^ CD64^+^MerTK^+^ CD11b^+^ SiglecF^−^ Trem2^hi^) populations were sorted from infected mice at 4 weeks post-infection by FACS cell sorting. For the coculture experiments, Trem2^l^° or Trem2^hi^ IM cells, were added to CFSE - labeled naïve CD4^+^ T cells at a 2:3 ratio. After 3 days, the CSFE signal of labeled cells was detected by FACS Canto II (BD Biosciences, San Jose, CA, USA).

### Cell sorting and scRNA-Seq

After incubation with Percp-Cy5.5 anti-mouse CD45 antibody (BD) and Ghost Dye™ Red 780 (Tonbo), CD45^+^ cells from lung cell suspensions pooled from three mice were sorted on a BD FACS ARIA II cell sorter.

To ensure the quality of samples, automated cell counting and sample quality tests were performed (Countess® II Automated Cell Counter with AO/PI reagent) after preparation of a single cell suspension. Single-cell libraries were constructed using Chromium Controller, Single Cell 5’ Library, and Gel Bead Kit (10x Genomics), and the quality of each library was assessed by Agilent 4200, then sequenced via the Illumina Novaseq platform. The Cell Ranger (version 3.0.2) was applied to demultiplex and align reads using mouse GRCm38/mm10 as a reference genome, and was also used to generate gene-barcode matrices. To combine data from all six samples, the aggr pipeline of Cell Ranger was employed.

### Quality control and scRNA-seq analysis

Quality control and unsupervised clustering analysis were implemented in Seurat (version 3.1.2) [17]. Cells with a total number of genes between 200 and 4000, and in which mitochondrial genes accounted for less than 20% of total UMIs, were included. In addition, we retained genes that were detected in at least three cells. After normalizing the raw counts of genes, the highly variable genes were calculated using the FindVariableFeatures function, and the top 2000 variable genes were used to perform PCA dimensional reduction. The JackStraw function and ElbowPlot function were applied to determine the principal components (PC). We used the function FindClusters to cluster cells, and set the resolution between 0.2 and 0.6. Then, a t-SNE plot, a non-linear dimensional reduction method, was used to visualize the data. Each cluster was annotated via canonical lineage markers. To remove the cell doublet, we used Scrublet to exclude doublets [18]. Each cell was calculated from the doublet score using default parameters, and clusters that contained more than 20% doublet cells were removed based on a cluster-level approach.

The clusters were annotated by SingleR with Immunological Genome Project (ImmGen) as a reference database and were corrected manually in accordance with canonical markers and a Seurat-based marker gene list.

To accurately annotate each cell type, a combination of a Seurat-based cluster-specific marker gene list, canonical markers (table S2), and SingleR were used to define cell clusters.

### Differentially expressed genes and gene set enrichment analysis

The FindMarkers function was utilized to calculate the DEGs between clusters based on a Wilcoxon rank sum test. We obtained only DEGs with a logFC > 0.25 that were expressed in more than 25% of cells in the cluster. DAVID website was used to perform the gene set enrichment analyses (https://david.ncifcrf.gov).

### Trajectory analysis of PAMs

To further verify the potential relations between Ly6C^+^ monocytes, PAMs, and IM-2 clusters in the mouse lung, those clusters were subjected to Monocle2 analysis to generate cellular trajectories. The top 500 variable genes were used to order cells in a semi-supervised manner. Dimensionality reduction and visualization were carried out via DDRTree and plot_cell_trajectory functions. The function of differentialGeneTest was used to generate the list of DEGs (q-value < 1 × 10^− 20^), and the plot_pseudotime_heatmap was applied for visualization. Gene modules were obtained by cutting the tree at k = 2. The trajectory was verified using diffusion maps in scater (version 1.14.0).

For transcription factor (TF) analysis, we adopted pySCENIC [19] to analyze the transcription factor regulons. The normalized expression matrix exported from Seurat was put into pySCENIC. We obtained the active TF along the cellular trajectory generated via Monocle2, and finally identified 19 TFs along the PAM trajectory.

### Dissecting cell-cell interactions

To investigate the cell-cell communication networks between mouse lung immune cells, we utilized CellPhoneDB to predict the LR pairs according to the expression levels of ligands and receptors in the given subcluster, and filtered those ligands or receptors that were expressed in less than 20% of the cells in the particular cluster. Since the database of LR pairs in CellPhoneDB is only for human samples, we converted the human database to mouse genes using the Mouse Genome Informatics Database [20].

### Bulk RNA-seq processing and data analysis

Both Trem2^l^° and Trem2^hi^ IM populations were sorted from *Pneumocystis*-infected mice at 4 weeks post-infection. Total RNA from sorted cell populations was reverse transcribed to cDNA, amplified, and prepared as sequencing libraries as previously described. Libraries were sequenced on a NextSeq 500 (Illumina).

## Results

### CD45^+^ cell composition of lung in mice infected with *Pneumocystis*

**Fig. 1:**
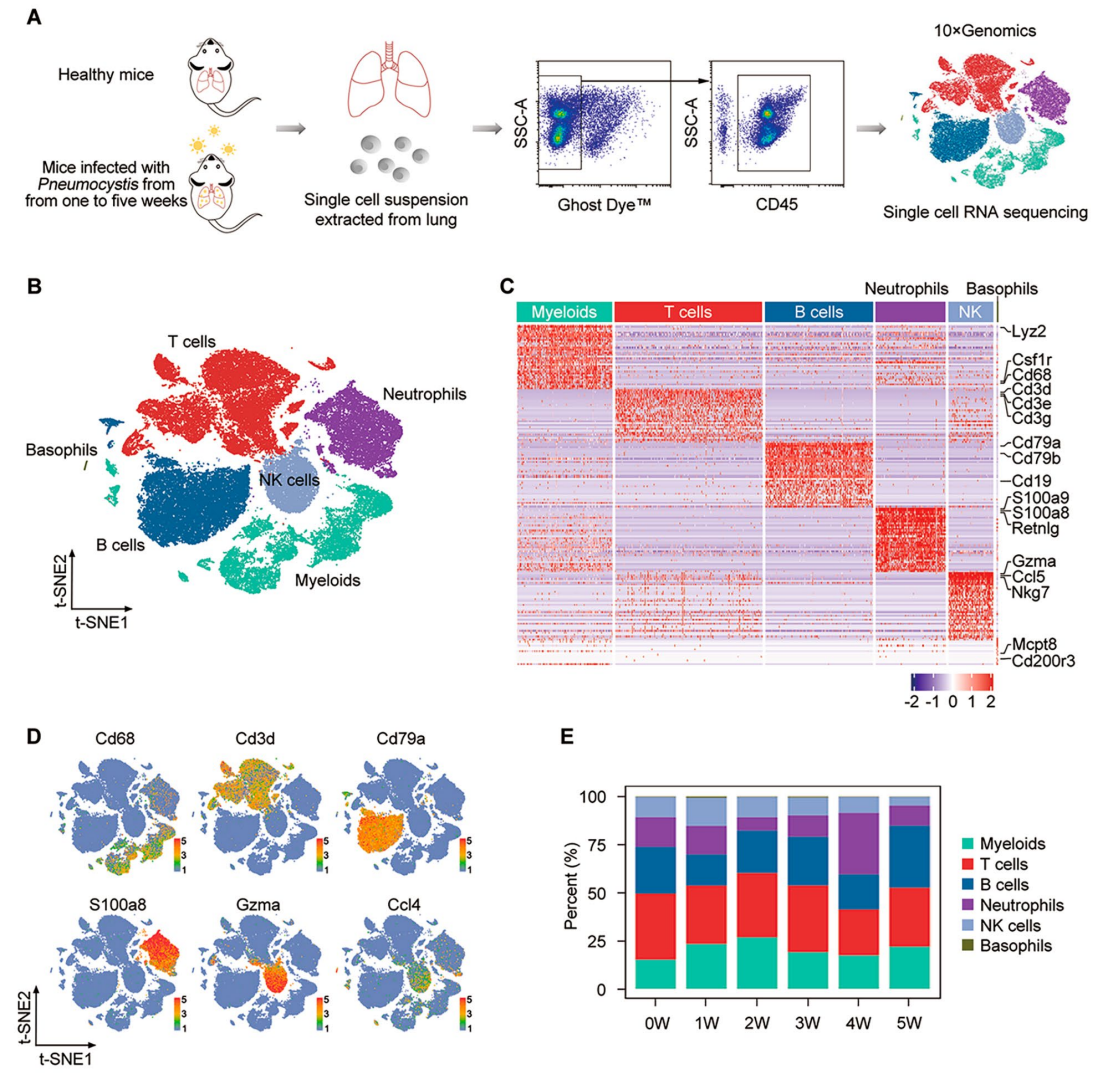
Identification of distinct cell types of CD45^+^ cells in mouse lung by scRNA-seq. **(A)** Overview of the experimental design. **(B)** Clustering of all 58,009 cells from all six samples via t-SNE visualization. **(C)** Heatmap showing the top genes in each subcluster of total cells. **(D)** t-SNE plot showing the expression of marker genes. **(E)** The proportion of myeloid cells, T cells, B cells, neutrophils, NK cells, and basophils across six samples

To fully elucidate the host immune environment during PCP, we first performed scRNA-seq on lung CD45^+^ cells using a well-established mouse model of *Pneumocystis* infection from 0 to 5 weeks (Fig. 1A). According to our previous studies, the *Pneumocystis* burden of this model would continuously increase until the third week, then decrease in the fourth week [8, 9]. Therefore, based on the change of *Pneumocystis* burden, we defined the period of 1 to 3 weeks post-infection as the acute infection stage, and the period of 4 to 5 weeks post-infection as the recovery stage (Fig. S1A). Transcriptional profiles of 63,500 total cells were obtained using the 10×Chromium platform and an average of 891 genes per cell were measured. After quality control and filtering, a total of 58,009 cells were ultimately included for subsequent analysis (Fig. S1B and table S3).

To determine cell identity, we obtained 15 clusters of CD45^+^ cells from six pulmonary samples at low-resolution, including myeloid cells, T cells, B cells, neutrophils, NK cells, and basophils (Fig. 1, B to D and Fig. S1 C to E). All clusters were comprised of cells from each sample (Fig. S1E) and the proportion of each cluster from each sample is shown in Fig. 1E. B cells were further re-clustered and three subclusters were identified, including naive B cells, plasma B cells and proliferating B cells (Fig. S1, F and G, table S4). The proportion of plasma B cells was significantly enriched in the lung in the 4-week post-infection (Fig. S1H). Collectively, the temporal dynamics of immune cells in the lung after *Pneumocystis* infection are clearly delineated.

### Dramatic reconstitution of myeloid compartments and emergence of PCP-associated macrophage following *Pneumocystis* infection

**Fig. 2:**
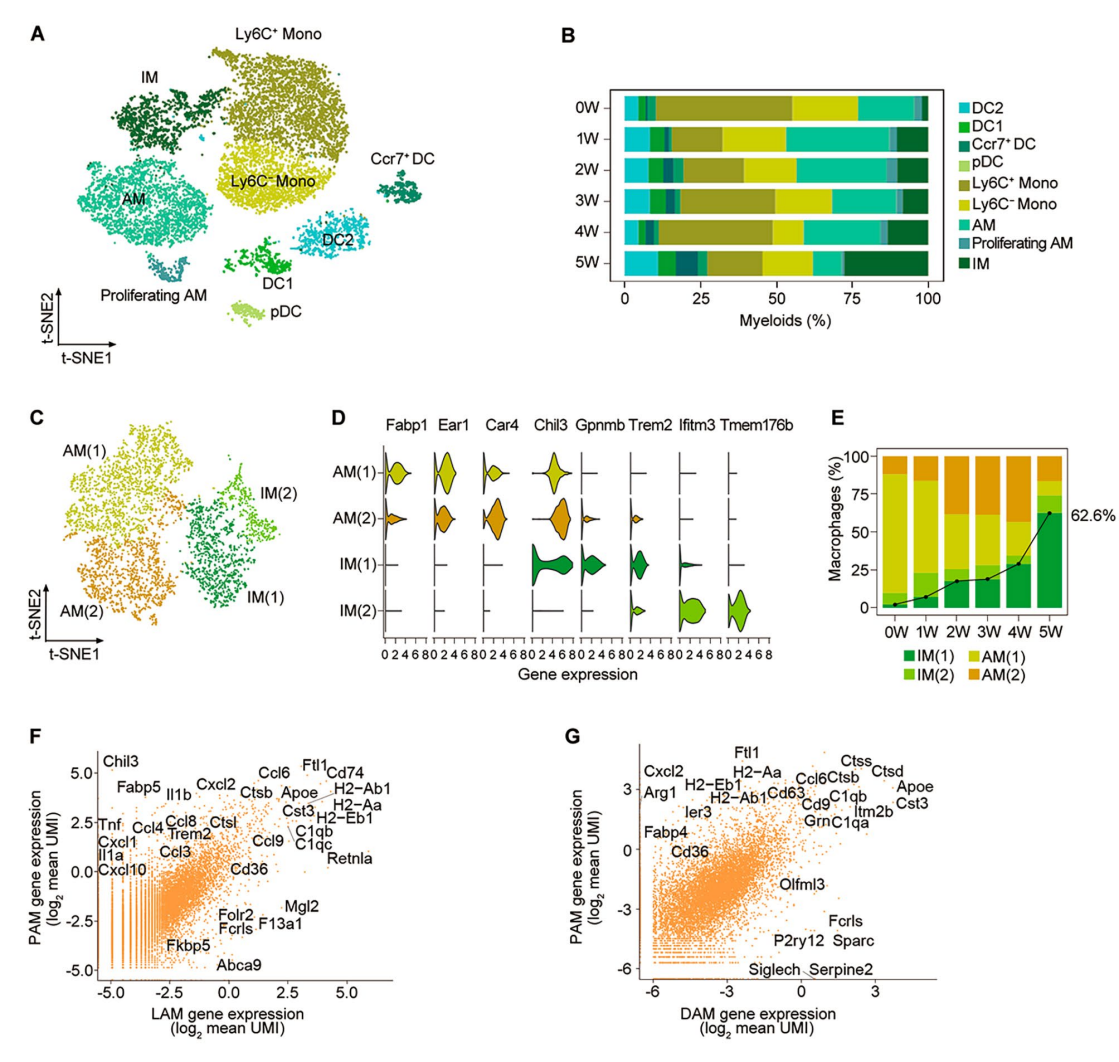
Focused analysis of lung myeloid cells. **(A)** Re-clustering revealed nine subclusters of myeloid cells. **(B)** The subtype frequency of myeloid cells from all six samples. **(C)** t-SNE plot of the subpopulation of macrophages. **(D)** Vinplot showing the expression of selected genes in the subpopulation of macrophages. **(E)** Cluster abundances of macrophage subclusters across uninfected and *Pneumocystis*-infected samples. **(F)** Scatterplot comparing the average UMI counts (log_2_ scale) of mouse PAM versus mouse LAM from the white adipose tissue of obese mice (Adhemar Jaitin al., 2019). **(G)** Scatterplot comparing the average UMI counts (log_2_ scale) of mouse PAM versus mouse DAM from the brains of Alzheimer’s disease mice (Keren-Shaulet al., 2017)

Myeloid cells are the first line of host immune system in combating the *Pneumocystis*, which were further re-clustered and yielded 9 clusters (Fig. 2A and table S4). Of these, four DC subsets (cDC2, cDC1, Ccr7^+^ DC, and plasmacytoid DC cells), two monocyte subsets (non-classical and classic monocytes), three macrophage subsets (alveolar, proliferating alveolar, and interstitial macrophages) were identified (Fig. S2, A and B). Among them, alveolar macrophages (AMs) increased at first, and then decreased in the process of *Pneumocystis* infection, suggesting that AMs mainly play a role in early host innate immunity against *Pneumocystis*. In addition, interstitial macrophages (IMs) accounted for only 2.0% of myeloid cells in the lung tissues of uninfected mice, but the proportion of IMs increased significantly to 27.6% in the fifth week after *Pneumocystis* infection (Fig. 2B). We repeated SingleR analysis using published RNA-seq data sets [21] of mouse lung macrophages, which conformed to the two major subtypes, AMs and IMs, as mentioned above (Fig. S2C). Heatmap demonstrated that the transcriptomes of both AMs and IMs were altered in response to *Pneumocystis* infection at different stages (Fig. S2D).

To elucidate macrophage heterogeneity, macrophages (AMs and IMs) were further re-clustered into four subgroups: AM-1 (Fabp1 and Ear1), AM-2 (Car4 and Chil3), IM-1 (Gpnmb and Trem2), and IM-2 (Tmem176b, Aif, Ifitm3) (Fig. 2, C and D, Fig. S2, E and F, and table S4). In particular, we observed a significant expansion of IM-1 in post-infection. Although IM-1 was detected in all of the samples, more than 99% of IM-1 cells were derived from PCP lungs, suggesting that this is a unique subset of macrophages related to pathogenesis in PCP (Fig. S2G). Thus, we inferred that the IM-1 cells might play a dominant role in PCP, and named IM-1 cells “PCP-associated macrophages” (PAMs). PAMs accounted for only 2.3% of macrophages from the lungs of uninfected mice, but the proportion of PAMs increased significantly to 62.6% in the fifth week following *Pneumocystis* infection (Fig. 2E and Fig. S2H).

### Characteristics of PCP-associated macrophage

Given the recent findings that Trem2 signaling of macrophages is conserved across different tissues [22], we next sought to evaluate whether Trem2 signaling of PAM cells could be found in other tissues and diseases. The results indicated that the transcriptomes of PAMs in our study were highly analogous to the genes in adipose tissue lipid-associated macrophages (LAMs) [22] and brain disease-associated microglia (DAMs) [23] (Fig. 2, F and G). Compared with LAMs, PAMs are highly expressed genes associated with cytokines and chemokines (e.g., Tnf, Il1b, Cxcl1, Cxcl10 and Ccl4). Likewise, apart from cytokines and chemokines, PAMs expressed more MHC-II molecules (e.g., H2-Aa, H2-Ab1, H2-Eb1) and tissue-specific genes (e.g., Cd36, Fabp4, ler3) compared with DAMs. The above results indicated that the PAM signature could be found in other tissues and diseases, but PAM cells also possessed its unique features induced by *Pneumocystis*.

**Fig. 3:**
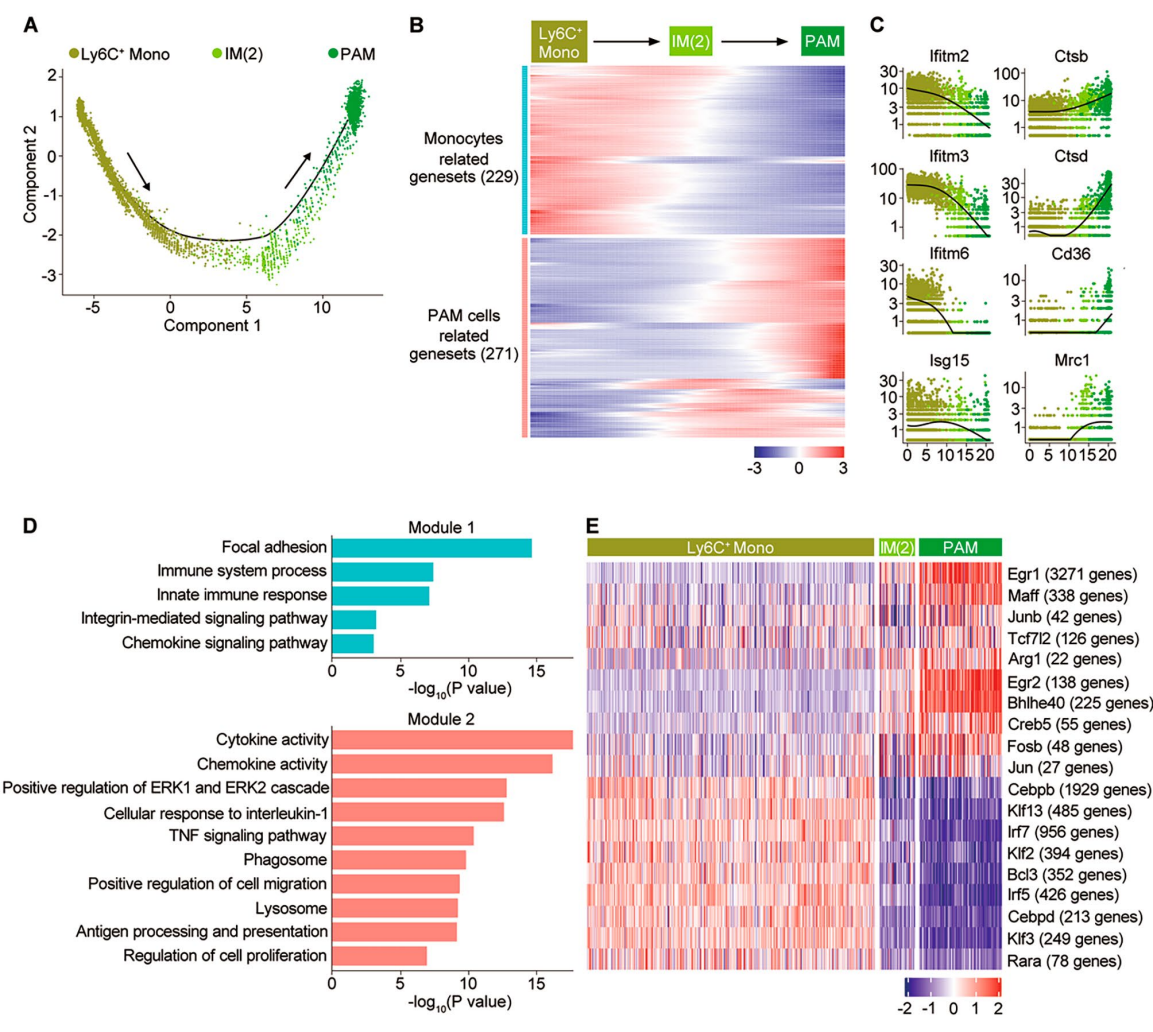
Trajectory analysis of PAM cells. **(A)** Pseudotime analysis by Monocle2 reveals specific trajectories of PAMs. **(B)** Heatmap showing DEGs along monocyte-to-PAM trajectory and hierarchical clustering (k = 2), indicating the expression patterns along the trajectory. **(C)** Expression of selected genes in each module along with the trajectory of PAMs. **(D)** Pathways enriched by genes in module 1 and module 2. **(E)** SCENIC results for the Ly6C^+^ monocytes and IMs. Heatmap showing the area under the curve (AUC) scores of expression regulated by transcription factors

Trajectory analysis suggested that the Ly6C^+^ monocytes primarily aggregated on the pseudotime backbone, with IM-2 linking monocytes and PAM cells (Fig. 3A), which was also confirmed by diffusion map (Fig. S2I). To further characterize the phenotype of PAM, we defined two gene co-expression modules during the differentiation of monocytes to PAM cells (Fig. 3B and table S5). The genes in module 2 were PAM related, consisting of multiple cytokines, chemokines, MHC class II genes, and phagocytic genes (Fig. 3C). These terms included cytokine activity, regulation of ERK1/2 cascade, and TNF pathways, as well as phagosome, lysosome, regulation of cell migration and proliferation, whereas module 1 contained a group of genes in which the expression levels decreased along the differentiation trajectories (Fig. 3D). Finally, to identify potential transcriptional regulators of PAMs, regulons were defined as sets of genes co-expressed with known transcription factors along the differentiation trajectories of PAM cells. 19 genes were identified as active transcription factors along the trajectory of PAMs, of which 10 transcription factors were upregulated during the differentiation from monocytes to PAMs, including Egr1, Maff, Junb, Tcf7l2, Arg1, Egr2, Bhlhe40, Creb5, Fosb, and Jun (Fig. 3E). Future experiments are needed to confirm the role of transcription factors in the process of monocyte differentiation into PAMs.

### Effector CD4^+^ T cells are strongly enriched after *Pneumocystis* infection

**Fig. 4:**
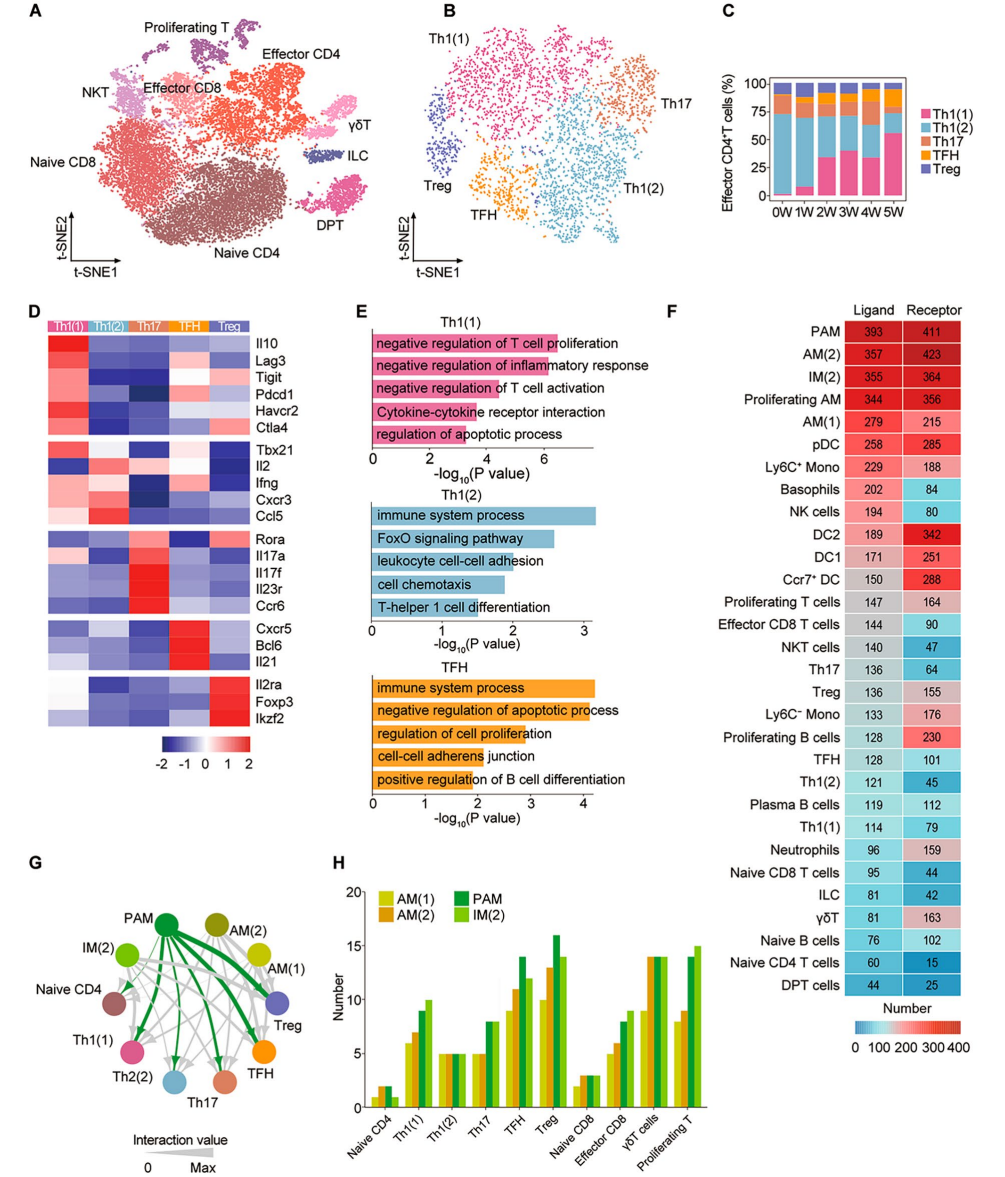
Focused analysis of lung T cells. **(A)** Re-clustering revealed nine T-cell subclusters. **(B)** t-SNE plot of the subpopulation of effector CD4^+^ T cells. **(C)** The composition of effector CD4^+^ T cells across all samples. **(D)** Heatmap showing the selected genes in each cluster of effector CD4^+^ T cells. **(E)** The enriched pathways of Th1-1, Th1-2, and TFH, respectively. **(F)** Heatmap showing the number of significant LR pairs. **(G)** The interactions between macrophage and CD4^+^ T cell subsets. The ligands of PAMs are marked in green and the others in gray. The arrow width is the sum of interaction values between two clusters. **(H)** The number of LR pairs from macrophages to T-cell clusters

We detected 18,310 T lymphocyte cells, NKT cells, and innate lymphoid cells (ILCs), which were clustered into nine subsets and distinguished as: Naive CD4^+^ T cells, Naive CD8^+^ T cells, Effector CD4^+^ T cells, Effector CD8^+^ T cells, γδT cells, double positive T cells (DPT cells), NKT cells, proliferating T cells and ILCs (Fig. 4, A, Fig. S3, A and B and table S4). Of these, naive CD4^+^ and CD8^+^ T cells showed a gradually decreasing trend, while effector CD4^+^ T cells significantly increased during the process of *Pneumocystis* infection (Fig. S3C). In addition, the transcriptomes of CD4^+^ T cells were altered during *Pneumocystis* infection (Fig. S3D).

To address the transcriptional states of CD4^+^ T cells in *Pneumocystis*-infected mice, we bioinformatically isolated and re-clustered effector CD4^+^ T cells. A total of five clusters were derived following Seurat clustering (Fig. 4B and table S4). The most abundant subsets were Th1(1) and Th1(2) cells (Fig. 4C). Specifically, Th1(1) and Th1(2) were associated with abundant Th1-related cytokines (Ifng, Cxcr3, Ccl5). Th1(1) was characterized by enrichment of IL-10 and inhibitory receptors, such as Lag3, Tim-3, and Tigit, indicating an immunoregulatory role. Other subsets of effector CD4^+^ T cells contained Th17 cells (Il17a, Tmem176a, Tmem176b), T follicular helper (TFH) cells (CXCR5, BCL6, IL21), and Treg cells (Il2ra, Foxp3, Ikzf2) (Fig. 4D).

Notably, over 99% of Th1(1) and TFH cells were derived from PCP lungs, suggesting the possibility of a link with PCP pathogenesis (Fig. S3, E and F). Since Th1(1) cells were highly specialized in IL-10 production, we calculated the make-up of IL-10 producing cells and found that Th1(1) was the most dominant source of IL-10, while plasma B cells and IM(2) cells were the main IL-10 producing cells among B cells and myeloid cells. These clusters all showed a continuously increasing trend during the process of *Pneumocystis* infection (Fig. S3G). In addition, a strong correlation between the proportion of TFH cells and plasma B cells was detected (Fig. S3H), suggesting the role of TFH in producing *Pneumocystis*-specific antibodies.

Next, to explore the function of effector CD4 subpopulations, we performed GO enrichment analyses. First, we found that the upregulated transcripts in Th1(1) cells were enriched for negative regulation of T cell proliferation and activation, cytokine-cytokine receptor interaction, and regulation of apoptotic processes, supporting the possibility that Th1(1) cells might play an anti-inflammatory and immune regulation role in PCP, especially during the recovery period. Second, Th1(2) cells had increased expression of transcripts associated with immune system process, FoxO signaling pathway, leukocyte cell-cell adhesion, cell chemotaxis, and Th1 cell differentiation. Third, TFH cells were enriched for regulation of cell proliferation, cell-cell adherens junction, and positive regulation of B-cell differentiation, which related to the functions of migration to lymphatic follicles and promoting B cell differentiation (Fig. 4E). These results illustrated that the subclusters of effector CD4^+^ T cells both underwent marked expansion and functional reprogramming during PCP pathogenesis.

### Trem2^hi^ interstitial macrophages accumulating in *Pneumocystis* infected lungs

To investigate the interactions among the lung immune cells, we calculated the score of ligand-receptor (LR) pairs. A dense intercellular communication network was revealed in each cell type. Notably, macrophages harbored the highest ligand numbers, with PAMs, AM(2), IM(2), proliferating AMs, and AM(1) harboring 393, 357, 355, 344, and 279, respectively (Fig. 4F). The number of L-R pairs and the sum of the interaction score between PAMs and CD4^+^ T cell subsets were higher than those of other macrophage subsets (Fig. 4, G and H). Taken together, we found that PAMs were the core of cell communication in lung immune cells, and also the key cluster for the interaction between macrophages and effector CD4^+^ T cells.

**Fig. 5:**
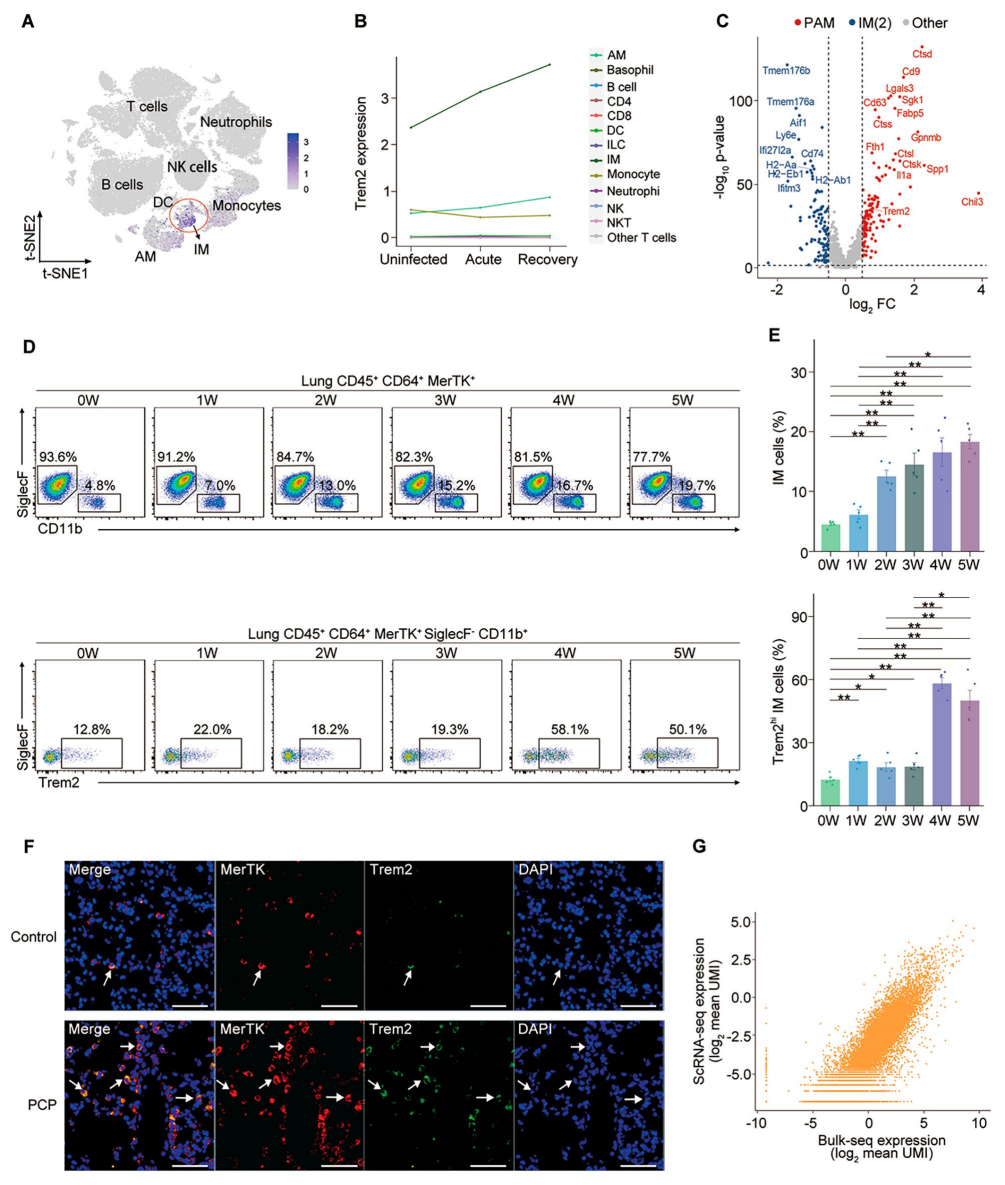
Validation of surface markers for PAM. **(A)** The expression of Trem2 in immune cells. **(B)** The expression of Trem2 in immune cells from uninfected and *Pneumocystis*-infected mice. **(C)** Volcano plot showing DEGs between PAMs and IM-2 (*P*-value < 0.05, log_2_FC > 0.5). **(D)** Representative flow cytometric plots of IMs (CD45^+^ CD64^+^ MerTK^+^ SiglecF^−^ CD11b^+^) (upper panel) and Trem2^hi^ IMs (CD45^+^ CD64^+^ MerTK^+^ SiglecF^−^ CD11b^+^ Trem2^hi^) (lower panel) from uninfected and *Pneumocystis*-infected mice, for which quantification is shown in (H). **(E)** Percentage of IMs among macrophages (upper), and Trem2^hi^ IMs among IMs (lower) in lungs from PCP and control groups, respectively (*n* = 5 per group). Bars represent mean ± SEM. *P* values were calculated by Mann-Whitney tests (* *P* ≤ 0.05; ** *P* ≤ 0.01). **(F)** Representative immunofluorescence images of 50-µm-thick lung sections from uninfected and *Pneumocystis*-infected mice (5-weeks post-infection) (DAPI [blue]; MerTK [red], Trem2 [green]). **(G)** Scatterplot comparing transcriptomes of PAMs measured by bulk sequencing versus scRNA-seq (Spearman correlation coefficient 0.88, *P* < 0.001)

Given the potential important role of PAMs in PCP, we sought to determine the surface markers of PAMs and we found that IM (CD45^+^ CD64^+^ MerTk^+^ SiglecF−) was the major cell population responsible for the Trem2 expression pattern after *Pneumocystis* infection (Fig. 5A). In addition, the expression of Trem2 in IMs was significantly increased after *Pneumocystis* infection, especially in the recovery stage (Fig. 5B). The differentially expressed genes (DEGs) showed low and high Trem2 mRNA expression (Trem2^l^° and Trem2^hi^, respectively) can be used to distinguish two IM subsets (Fig. 5C). We validated the presence and expansion of Trem2^hi^ IMs in PCP by flow cytometry (Fig. 5, D and E, Fig. S4A) and tissue staining (Fig. 5F). In addition, their proportions of macrophages quantified by flow cytometry correlated significantly with those detected by scRNA-seq (Pearson *r* = 0.84) (Fig. S4B). To fully validate these findings, we isolated PAM cells by flow cytometry and performed bulk sequencing. The results indicated that the transcriptomes of PAMs measured by bulk sequencing correlated significantly with scRNA-seq (Pearson *r* = 0.89) (Fig. 5G, table S6). This panel of surface markers could be used to identify PAM cells in order to further explore their functional role.

**Fig. 6:**
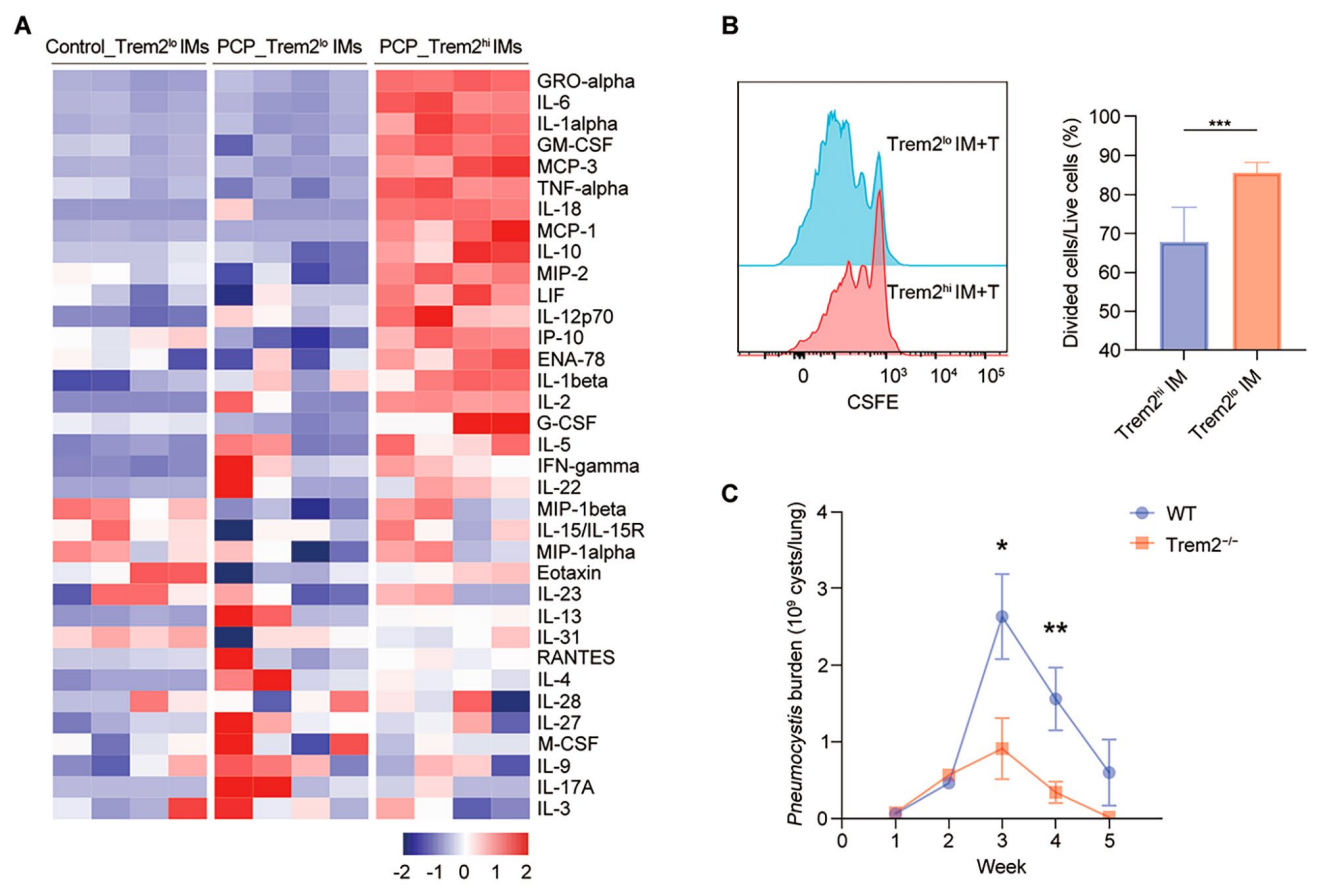
Functional properties of PAM. **(A)** Heatmap showing the relative abundance of the indicated proteins secreted by Trem2^l^° IMs sorted from uninfected or PCP mice, and Trem2^hi^ IMs sorted from PCP mice (4-weeks post-infection) (*n* = 4). **(B)** Flow cytometric plots showing proliferation of CFSE-labeled T cells after 3 days co-culture with Trem2^hi^ and Trem2^lo^ IMs (left); mean percentage of proliferating cells in cultures of each group (right). **(C)** The *Pneumocystis* burden in WT mice and Trem2^−/−^ mice from 0 to 5 weeks after infection. The results were from five mice per group per timepoint

To explore the distinct function of IM subsets, we quantified a multiplex protein in the supernatants of IMs. On the whole, the proteome profiling of Trem2^l^° IM cells from *Pneumocystis*-infected mice was comparable to that of IMs derived from uninfected mice, while Trem2^hi^ IMs exhibited distinct features, and were characterized by elevated pro-inflammation cytokines (IL-6, IL-1α, TNF-α, IL-18, IL-12p70, IL-1β, IL-2, IL-5, IFN-γ, IL-22), immunoregulatory cytokines (IL-10), chemokine secretory profile (GRO-α, MCP-3, MCP-1, MIP-2, IP-10, ENA-78, Ccl5), and proteins involved in cell growth and differentiation (GM-CSF, LIF, G-CSF) (Fig. 6A and Fig. S4C). Therefore, it could be inferred that the function of Trem2^l^° IMs in PCP was similar to IMs in uninfected mice, while Trem2^hi^ IMs induced by *Pneumocystis* possessed different proteome profiles. Moreover, we sought to determine the regulatory effect of IM on T cell proliferation, Trem2^hi^ and Trem2^lo^ IM were co-cultured with naive T cells, which found that Trem2^hi^ IM significantly inhibited T cell proliferation (Fig. 6B).

To investigate the role of Trem2 in PCP, WT and Trem2^−/−^ mice were infected with *Pneumocystis*. the *Pneumocystis* burden was significantly decreased in Trem2^−/−^*Pneumocystis*-infected mice compared to WT *Pneumocystis-*infected mice at Week 3 and 4 (Fig. 6C). In addition, fewer inflammatory cells around the pulmonary vessels and adjacent alveoli of Trem2^−/−^ mice compared with WT mice (Fig. S4D). Collectively, this indicate Trem2 inhibited the clearance of *Pneumocystis*.

## Discussion

PCP is an important cause of morbidity in immunocompromised patients, with an increasing rate of morbidity and highly mortality in non-HIV patients [24]. We previously characterized Th9 cells, B10 cells, and macrophages in PCP based on bulk cell sequencing [8–10, 25] but little is known about the transcriptome dynamics in immune cells from different timepoints during *Pneumocystis* infection. In this study, the transcriptome data of more than 58,000 individual CD45^+^ cells from mouse lung tissue from multiple timepoints during the course of PCP were generated using 10×Genomics, establishing a rich resource for comprehending multidimensional characterization of immune cells in PCP. Our results identified a disease-specific IM cell subset, clarified its potential function, and constructed a ligand-receptor network between PAM and CD4^+^ T cells.

These findings show insights into the characteristics and functions of macrophage subsets in PCP. The resident tissue macrophages in the lungs are very heterogeneous, and they can be classified as two types: AMs, residing in the alveolar spaces, and IMs, which differentiate from blood monocytes and are located near the larger airways or in the lung interstitium [14]. Although much attention has been focused on AMs in PCP, we identified IMs that were strongly enriched after *Pneumocystis* infection. TLR4 ligand lipopolysaccharide and TLR9 ligand CpG-DNA could promote the enrichment of IMs in the lung, while influenza A virus or *Staphylococcus*, ligands to TLR-5 and 7/8, failed to induce the expansion of IMs [26]. In this study, we found that *Pneumocystis* was one of the stimuli inducing the accumulation of IMs in the lungs, although the specific TLR of *Pneumocystis* remains unclear.

Further dissecting the heterogeneity of IMs, we uncovered a unique subset of IM associated with host immune response to *Pneumocystis*, which was characterized by high Trem2 expression. However, the phenotype and function of IM in PCP have never been investigated. Herein, we found the emergence of Trem2^hi^ IMs in PCP, especially in the recovery stage, not only using scRNA-seq, but also via several analyses such as flow cytometry, immunostaining and bulk sequencing. Of note, different protein expression profiles were found between IM subsets, and Trem2^hi^ IMs were endowed with a superior capacity to secrete multiple proteins. Some of the proteins described in our study, including IL-6, IL-1, TNF-α, and IL-10, have already been reported to play important roles in the function of IMs, and most of them (e.g. IL-6, TNF-α, IP-10, and IL-12p70) [27–30] have been reported to be involved in PCP. These results demonstrate that *Pneumocystis* induces the emergence of Trem2^hi^ IMs, and this type of IMs could drive inflammatory and anti-inflammatory cytokine production within the alveolar space during *Pneumocystis* infection. Understanding the origin of Trem2^hi^ IM, and the related intracellular signaling pathways will require additional studies, to fully elucidated its effect on the clearance of *Pneumocystis*.

In addition to macrophages, other immune cell subsets are also worth noting in host immune responses against *Pneumocystis*. In addition to myeloid cells, we also investigated the dynamic changes in lymphocytes. Although many studies have been dedicated to examining the role of CD4 cells in controlling *Pneumocystis* infection, it remains unclear which subset of T cells is necessary for this control. Interestingly, we observed an accumulation of Th1-1 cells in PCP which were marked by the expression of abundant IL-10, as well as inhibitory receptors (Lag3, Pdcd1, Ctla4, Tim3 and Tigit), indicating an immune regulatory and immunosuppressive role in PCP. Consistent with the previous findings, Th1 and Tr1 cells are implicated as being the predominant sources of IL-10 during chronic viral and parasitic infections. An increase in a similar subset has also been observed in lung fibrosis and could attenuate lung inflammation and fibrosis [31]. Since Trem2^hi^ IMs was shown to inhibit T cell proliferation via the co-culture experiments, we next focused on the interaction between macrophages and T cells by analyzing ligand-receptor pairs. Unexpected and complex interactions between the immune cells were delineated in our work. PAMs harbored the highest ligand numbers and the highest interaction score with CD4 cells, suggesting that PAMs play a key regulatory role in cell communication and have a potential regulatory effect on T cells.

Finally, our findings suggested that Trem2 participated in the clearance of *Pneumocystis*. An emerging role for Trem2 in regulating immune functions of macrophages in infectious diseases, including bacterial, viral, and parasitic infections [32–34]. In mycobacterial infections, Lizasa et al. indicated that Trem2 suppressed the activation of macrophages and ultimately lead to mycobacterial evasion of host immunity [35]. Similar to our results, Upadhyay et al. observed a rapid recruitment of TREM2^+^ macrophages upon SARS-CoV-2 infection, which are the predominant source of inflammatory cytokines [36]. In the context of porcine reproductive and respiratory syndrome virus (PRRSV) infection, Zhu et al., indicated that the Trem2 silencing restrained the replication of PRRSV and triggered an early proinflammatory response [33]. However, the functional role and mechanism of Trem2 during fungal infection in interstitial macrophages remain unclear. In our study, Trem2 knockdown contributed to the reduced *Pneumocystis* burden and lung injury, which underlines the possibility that targeting Trem2 could be a new approach for control of *Pneumocystis* infection beyond neurodegenerative disease, obesity-related metabolic syndrome, and cancer.

Our study is limited by its relatively small sample size. However, we primarily aimed to dissect the transcriptome dynamics across the progression of *Pneumocystis* infection at a high resolution. Moreover, IM cells and their two predominant subtypes in murine lungs were clearly distinguished by scRNA-seq. Furthermore, we performed flow cytometry to verify the increase of Trem2^hi^ IMs in PCP and performed immunofluorescence to confirm the co-expression of Trem2 and MerTK, which strengthen the reliability of our transcriptional data for immune cells during PCP.

Taken together, our comprehensive profiling of immune cells from different timepoints after *Pneumocystis* infection at the single-cell level tracked the dynamic and diverse changes in the transcriptomes of CD45^+^ cells in PCP. Our work identified multiple lineages of myeloid and lymphoid cells and specific PCP-associated subsets, providing a framework for further investigation into PCP’s cellular and molecular basis, which could lead to the discovery of novel biomarkers and therapeutic targets.

### Electronic supplementary material

Below is the link to the electronic supplementary material.

**Supplementary Material 1:Fig. S1. Quality control and annotation of mouse lung CD45**^**+**^**cells, related to Fig. 1. (A)** The *Pneumocystis* burden from 0 to 5 weeks after infection. The results were from three mice per timepoint. **(B)** Violin plots of the number of genes (left), number of UMIs (middle) and proportion of mitochondrial genes (right) across 58,009 lung immune cells. **(C)** Annotation of clusters identified with low-resolution from all samples. **(D)** Heatmap showing the expression of marker genes in clusters identified with low-resolution. **(E)** t-SNE plots showing the clustering of cells from each sample. **(F)** t-SNE plot of the subpopulation of B cells. **(G)** Heatmap showing the top genes in B-cell subclusters. **(H)** The abundance of B-cell subsets

**Supplementary Material 2**: **Fig. S2. Mouse lung myeloid cells, related to Fig. 2. (A)** Selected gene expression in myeloid cells. **(B)** Heatmap showing the top genes in each subcluster of myeloid cells. **(C)** AMs and IMs were annotated by SingleR using the reference data (GSE94135). **(D)** Heatmap highlighting DEGs in IMs and AMs from different stages of *Pneumocystis* infection. **(E)** Heatmap showing the top 20 genes in each subcluster of macrophages. **(F)** The composition of macrophages in all samples. **(G)** The proportion of PAMs from the six samples. **(H)** The clustering of macrophage cells from each sample. **(I)** Diffusion map revealed a trajectory beginning with Ly6C^+^monocytes and progressing to terminally differentiated PAMs

**Supplementary Material 3**: **Fig. S3. Mouse lung T cells, related to Fig. 4. (A)** Selected gene expression patterns in T cells. **(B)** Vinplot showing the expression of selected genes in the subcluster of T cells. **(C)** The abundance of T cell subsets. **(D)** Heatmap highlighting DEGs in naive and effector CD4^+^ T cells from different stages of *Pneumocystis* infection. **(E)** Heatmap showing the expression of canonical markers in effector CD4^+^ T cell subclusters. **(F)** The clustering of effector CD4^+^ T cells from each sample. **(G)** The composition of cells expressing IL-10. **(H)** The correlation between the change of proportion of Tfh and plasma B cells (Spearman correlation coefficient 0.89, *P* = 0.03)

**Supplementary Material 4**: **Fig. S4. Mouse lung PAMs, related to Figs. 5 and 6. (A)** Gating strategy used for analysis of IMs. **(B)** The proportions of PAMs quantified by flow cytometry versus scRNA-seq (Pearson r = 0.84). **(C)** The quantification of selected proteins in culture supernatants of Trem2^l°^ IMs sorted from uninfected or PCP mice, and Trem2^hi^ IMs sorted from PCP mice (n = 4) by Luminex assay. Data are mean ± SEM. *P* values were calculated by Mann-Whitney tests (*P ≤ 0.05). **(D)** Hematoxylin and eosin (H&E)-stained histological features of lungs in WT mice and Trem2^-/-^ after *Pneumocystis* infection from 1 to 4 weeks

**Supplementary Material 5**: **Table S1. Antibodies used in the study**

**Supplementary Material 6**: **Table S2. Canonical markers list**

**Supplementary Material 7**: **Table S3. Information about quality controls for scRNA-seq.** This table provides information regarding the quality control in our scRNA-seq for all six samples, and shows the data of Pre-QC (before performing quality control) and Post-QC (after performing quality control)

**Supplementary Material 8**: **Table S4. Marker gene list.** A list of marker genes from the cell clusters

**Supplementary Material 9**: **Table S5. Two differential gene modules and PAM trajectory.** A list of genes associated with two co-expression modules of DEGs along the PAM pseudotemporal trajectory (Fig. 4B)

**Supplementary Material 10**: **Table S6. Differentially expressed genes between Trem2**^**l°**^ **IM and Trem2**^**hi**^ **IM from bulk-seq**

## Data Availability

The data and codes associated with this work are available from the corresponding author upon request. scRNA-seq data of CD45^+^ cells from lung tissue with or without Pneumocystis infection can be found in the Gene Expression Omnibus (GEO) repository through accession number GSE157627.
