# Supplementary figures and images for: Dynamics of host immune responses and a potential function of Trem2hi interstitial macrophages in Pneumocystis pneumonia
Source: Respir Res. 2024 Feb 5;25:72. doi: 10.1186/s12931-024-02709-1 (PMC10845524; doi:10.1186/s12931-024-02709-1)

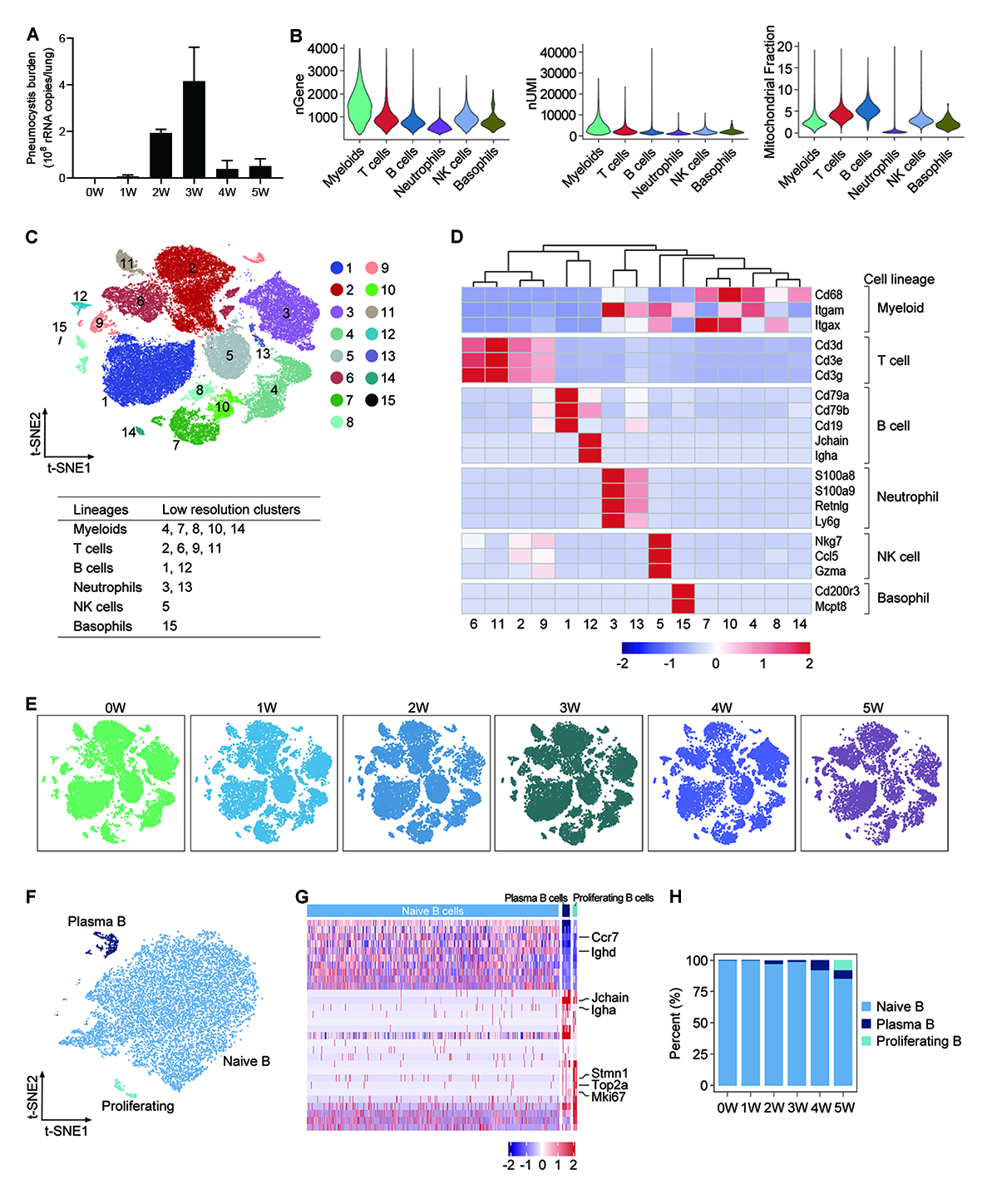

Supplement: Supplementary file 1 — Supplementary Material 1:Fig. S1. Quality control and annotation of mouse lung CD45+cells, related to Fig. 1. (A) The Pneumocystis burden from 0 to 5 weeks after infection. The results were from three mice per timepoint. (B) Violin plots of the number of genes (left), number of UMIs (middle) and proportion of mitochondrial genes (right) across 58,009 lung immune cells. (C) Annotation of clusters identified with low-resolution from all samples. (D) Heatmap showing the expression of marker genes in clusters identified with low-resolution. (E) t-SNE plots showing the clustering of cells from each sample. (F) t-SNE plot of the subpopulation of B cells. (G) Heatmap showing the top genes in B-cell subclusters. (H) The abundance of B-cell subsets [file 12931_2024_2709_MOESM1_ESM.tif]

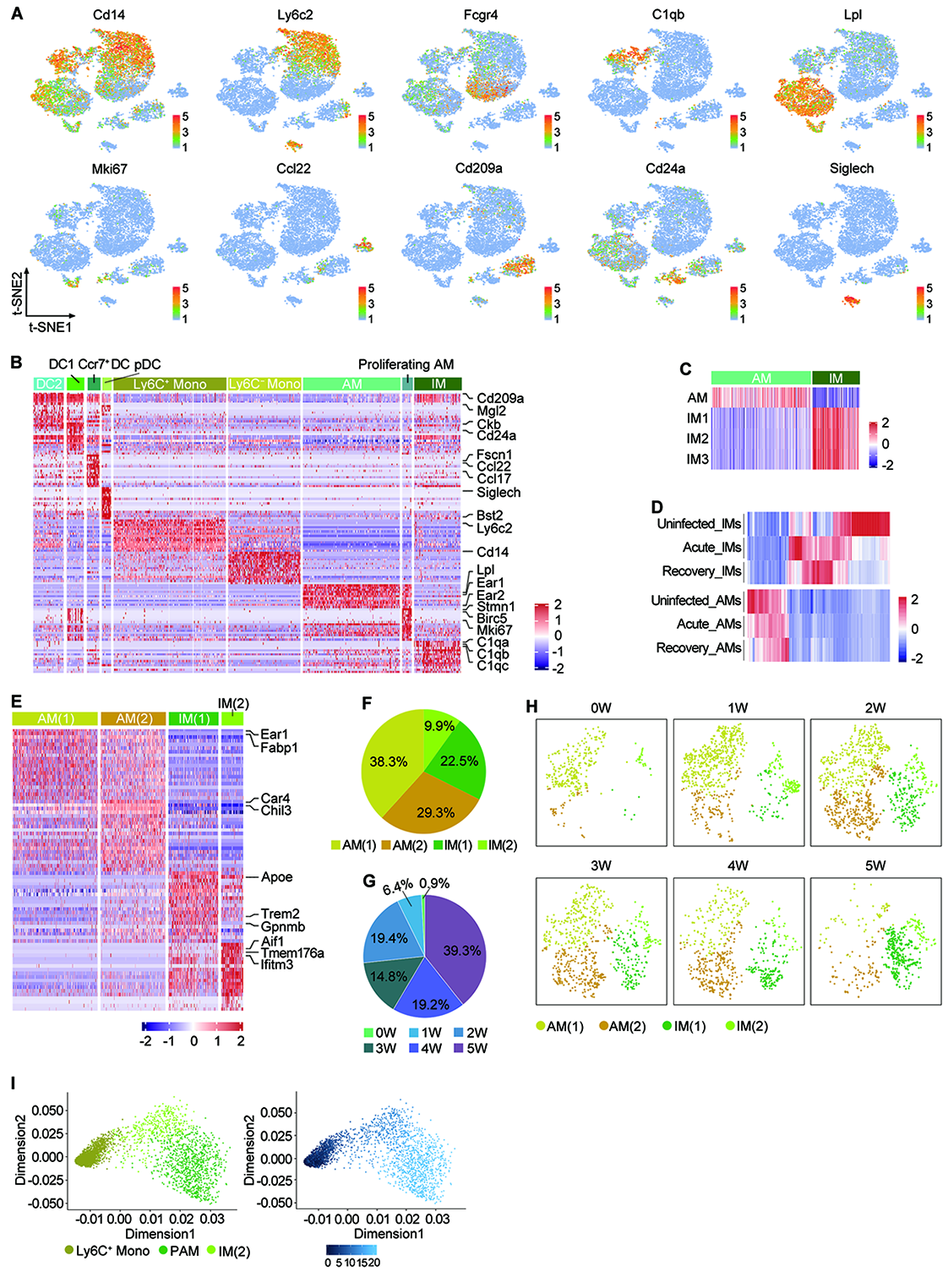

Supplement: Supplementary file 2 — Supplementary Material 2: Fig. S2. Mouse lung myeloid cells, related to Fig. 2. (A) Selected gene expression in myeloid cells. (B) Heatmap showing the top genes in each subcluster of myeloid cells. (C) AMs and IMs were annotated by SingleR using the reference data (GSE94135). (D) Heatmap highlighting DEGs in IMs and AMs from different stages of Pneumocystis infection. (E) Heatmap showing the top 20 genes in each subcluster of macrophages. (F) The composition of macrophages in all samples. (G) The proportion of PAMs from the six samples. (H) The clustering of macrophage cells from each sample. (I) Diffusion map revealed a trajectory beginning with Ly6C+monocytes and progressing to terminally differentiated PAMs [file 12931_2024_2709_MOESM2_ESM.tif]

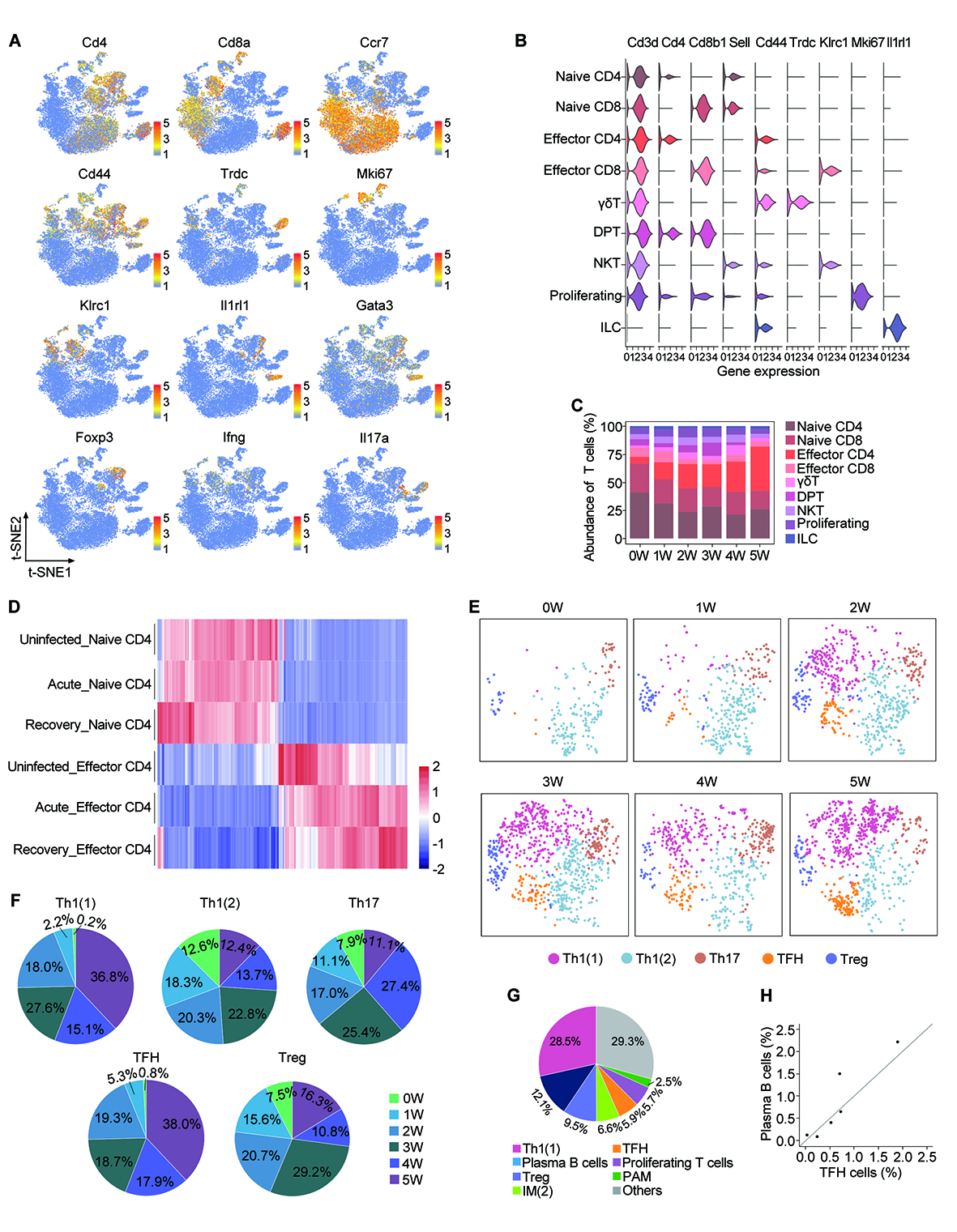

Supplement: Supplementary file 3 — Supplementary Material 3: Fig. S3. Mouse lung T cells, related to Fig. 4. (A) Selected gene expression patterns in T cells. (B) Vinplot showing the expression of selected genes in the subcluster of T cells. (C) The abundance of T cell subsets. (D) Heatmap highlighting DEGs in naive and effector CD4+ T cells from different stages of Pneumocystis infection. (E) Heatmap showing the expression of canonical markers in effector CD4+ T cell subclusters. (F) The clustering of effector CD4+ T cells from each sample. (G) The composition of cells expressing IL-10. (H) The correlation between the change of proportion of Tfh and plasma B cells (Spearman correlation coefficient 0.89, P = 0.03) [file 12931_2024_2709_MOESM3_ESM.tif]

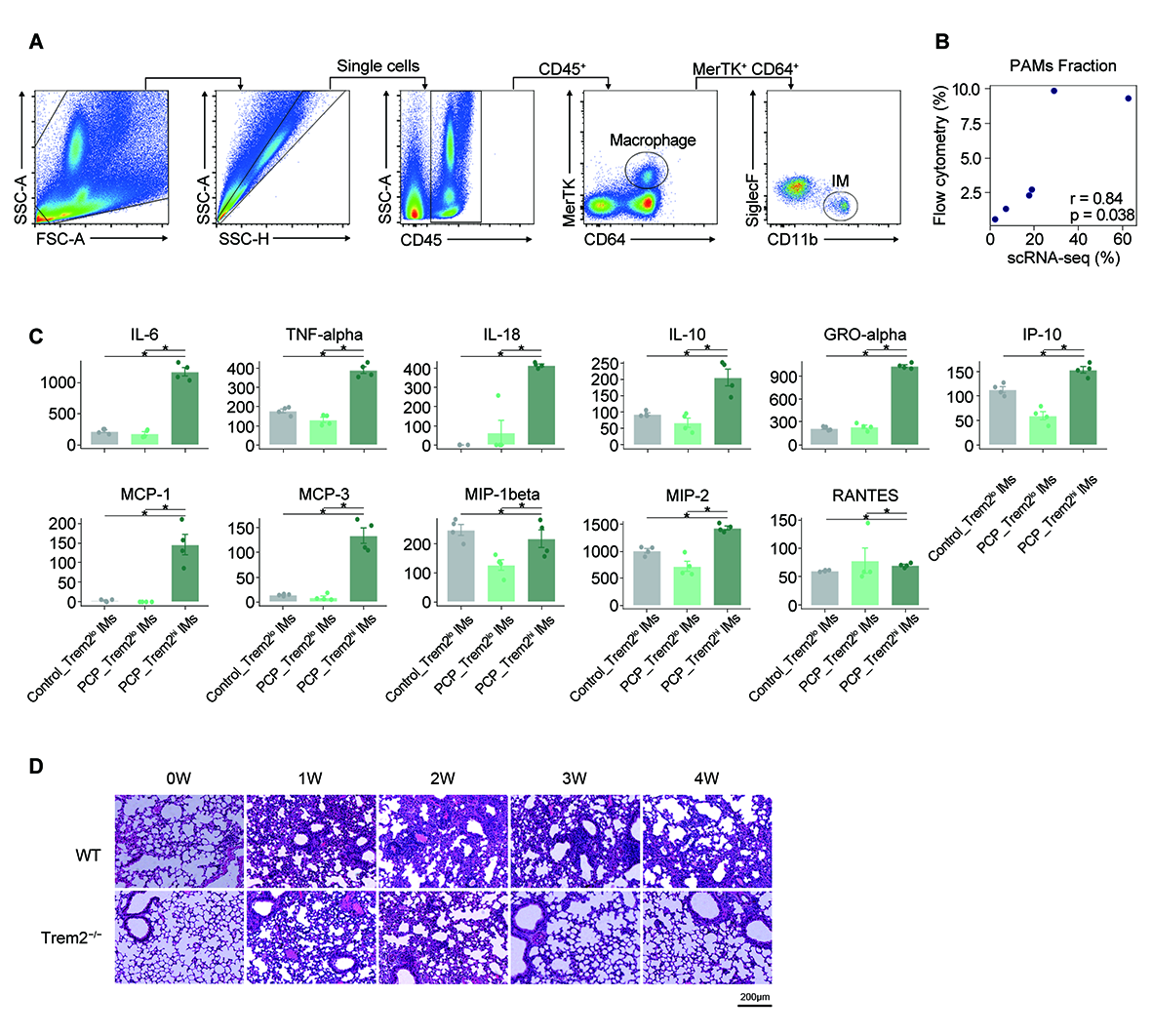

Supplement: Supplementary file 4 — Supplementary Material 4: Fig. S4. Mouse lung PAMs, related to Figs. 5 and 6. (A) Gating strategy used for analysis of IMs. (B) The proportions of PAMs quantified by flow cytometry versus scRNA-seq (Pearson r = 0.84). (C) The quantification of selected proteins in culture supernatants of Trem2l° IMs sorted from uninfected or PCP mice, and Trem2hi IMs sorted from PCP mice (n = 4) by Luminex assay. Data are mean ± SEM. P values were calculated by Mann-Whitney tests (*P ≤ 0.05). (D) Hematoxylin and eosin (H&E)-stained histological features of lungs in WT mice and Trem2-/- after Pneumocystis infection from 1 to 4 weeks [file 12931_2024_2709_MOESM4_ESM.tif]
